# Supplementary material for: Silicon Nanomaterials Enhance Seedling Growth and Plant Adaptation to Acidic Soil by Promoting Photosynthesis and Antioxidant Activity in Mustard (Brassica campestris L.)
Source: Int J Mol Sci. 2024 Sep 25;25(19):10318. doi: 10.3390/ijms251910318 (PMC11477286; doi:10.3390/ijms251910318)
Supplement: Supplementary file 1 [file ijms-25-10318-s001.zip › ijms-3199455-supplementary.pdf]

Supplementary Table S1: Soil physical and chemical properties before sowing of mustard seeds at two different location.

Soil types	AEZ (Agro Ecological Zone)	Physical properties			Chemical properties		
		% Sand	% Silt	% clay	pH ranges	EC mS/Cm	OM %
Field soil	AEZ-20 (Eastern Surma Kusiara Flood Plain) (24°54'30.4"N 91°47'17.5"E)	32.24	44.72	23.04	6.2 - 6.3	0.065	3.86
Acid soil	AEZ-22 (Northern and Eastern Piedmont Plain) (24°54'32.4"N 91°56'33.5"E)	71.2	23.64	6.12	4.5 - 4.8	0.042	3.06
